# Tracking Changes in Frontal Lobe Hemodynamic Response in Individual Adults With Developmental Language Disorder Following HD tDCS Enhanced Phonological Working Memory Training: An fNIRS Feasibility Study

**DOI:** 10.3389/fnhum.2020.00362

**Published:** 2020-09-10

**Authors:** Amy Berglund-Barraza, Fenghua Tian, Chandramallika Basak, John Hart, Julia L. Evans

**Affiliations:** ^1^School of Behavioral and Brain Sciences, The University of Texas at Dallas, Dallas, TX, United States; ^2^Department of Bioengineering, The University of Texas at Arlington, Arlington, TX, United States

**Keywords:** developmental language disorder, spoken word processing, functional near-infrared spectroscopy, memory updating and inhibition, prefrontal cortex, n-back, high definition transcranial direct current stimulation

## Abstract

**Background:** Current research suggests a neurobiological marker of developmental language disorder (DLD) in adolescents and young adults may be an atypical neural profile coupled with behavioral performance that overlaps with that of normal controls. Although many imaging techniques are not suitable for the study of speech and language processing in DLD populations, fNIRS may be a viable option. In this study we asked if fNIRS can be used to identify atypical cortical activation patterns in individual adults with DLD and track potential changes in cortical activation patterns following a phonological working memory training protocol enhanced with anodal HD tDCS stimulation to the presupplementary motor area (preSMA).

**Objective/Hypothesis:** The purpose of this study was two-fold: (1) to determine if fNIRS can be used to identify atypical hemodynamic responses in individual young adults with DLD during active spoken word processing and, (2) to determine if fNIRS can detect changes in hemodynamic response in these same adults with DLD following anodal HD tDCS enhanced phonological working memory training.

**Methods:** Two adult subjects with DLD (female, age 25) completed a total of two sessions of fNIRs working memory task prior to and following one session of a non-word repetition task paired with anodal HD tDCS (1.0 mA tDCS; 20 min) to the preSMA. Standardized *z*-scores of behavioral measures (accuracy and reaction time) and changes in hemodynamic response during an n-back working memory task for the two participants with DLD was compared to that of a normative sample of 21 age- and gender- matched normal controls (ages 18 to 25) prior to and following phonological working memory training.

**Results:** Individual standardized *z*-scores for each participant with DLD indicated that prior to training, hemoglobin response in the prefrontal lobe for both participants was markedly different from each other and normal controls. Following training, standard scores showed that the hemodynamic response for both participants moved within normal limits for ROIs.

**Conclusion:** These findings highlight the feasibility of fNIRS to establish individual differences in the link between behavior and neural patterns in single subjects with DLD, as well as track individual differences in changes in brain activity following working memory training.

## Introduction

Developmental language disorder (DLD), also referred to as Specific Language Impairment (SLI), is a neurodevelopmental disorder characterized by failure to master spoken and written comprehension and production in the absence of intellectual disability, hearing loss, or other medical conditions or syndromes known to cause language disorders. In addition to deficits in the acquisition and use of language, individuals with DLD have deficits in non-linguistic cognitive domain as well (Bishop, 2014). Although numbers vary slightly across countries, in the United States ∼ 7% of English-speaking school-aged children have DLD (Tomblin et al., 1997). The purpose of this study was two-fold: (1) to determine if fNIRS is sensitive to the presence of atypical hemodynamic responses in young adults with Developmental Language Disorder (DLD) during active spoken word processing and, (2) to determine if fNIRS is able to detect potential changes in hemodynamic response in adults with DLD following anodal HD tDCS enhanced phonological working memory training.

Although much of the research in DLD has focused on preschool and school-aged children, DLD is a long-term disorder that persists into adolescence and adulthood (Tomblin et al., 1992; Durkin and Conti-Ramsden, 2007; Catts et al., 2008; Conti-Ramsden, 2013). Historically, with some exceptions, the focus of most of the research in DLD has been on the earlier stages of language development, characterizing the deficit profile in preschool and elementary school-aged children and to a lesser extent adolescents and young adults. This was due in part to studies suggesting that some children initially diagnosed with DLD “appear” to achieve close-to-normal language skills at later ages, leading to the idea that these children were simply delayed in their acquisition of language (i.e., Rice et al., 2009). Large-scale longitudinal studies now show, however, that some individuals with DLD continue to exhibit significant language deficits into young adulthood (i.e., Borovsky et al., 2013; Brown et al., 2014; Haebig et al., 2017).

Importantly, studies that indicate DLD persists in adolescents and young adults have focused on the link between brain and behavior. Only by examining the relationship between behavioral performance and cortical activation patterns have these studies have been instrumental in demonstrating that some adolescents with a history of DLD may exhibit behavioral performance on both standardized tests and experimental tasks that “resembles” that of typically developing controls; yet despite this apparent overlap in behavioral performance, the underlying neural circuits that are engaged by these individuals with DLD differ qualitatively from those of their typically developing peers (Brown et al., 2014; Haebig et al., 2017).

This pattern that is emerging in DLD studies where behavioral performance falls at the low end of a normal continuum but the temporal and spatial cortical dynamics of language processing are qualitatively different from normal controls, is suggestive of an abnormal pattern that is distinct from those with normal language and may be a potential clinical marker of DLD in adults (e.g., Brown et al., 2014). A problem that has complicated researchers’ ability to address this question is limitations in some neuroimaging technologies. Advances in non-invasive functional neuroimaging technologies, including fMRI, MEG, EEG, and PET, have significantly improved the resolution of spatial and temporal brain imaging data resulting in profound changes in our understanding of brain structure and function for language and cognitive processing. However, our understanding of the link between brain structure and function for spoken language processing in general, as well as our understanding of the developmental changes in spoken language abilities in individuals with DLD has lagged. This is due, in part, to the methodological limitations of some of these technologies which include high sensitivity to motion artifacts, and the lack of methodological suitability for use in spoken language studies due to MRI scanner noise.

A second problem that has complicated researchers’ ability to characterize the brain behavior relationship in DLD has been the use of group research designs that rely on average group imaging data, an approach that obscures potential individual differences that may be relevant for the identification and/or differentiate of DLD and neurotypicals. Notably, as noted in Brown et al. (2014), group-averaged brain activation patterns show only the neuroanatomical components that are consistent across subjects, but obscures potentially critical individual differences. In particular, for clinical populations where there is high inter-individual variability, averaging across hemodynamic patterns may not be specifically representative of any one individual with the disorder in question. It has been argued that to gain a more detailed characterization of the relationship between brain and behavioral, it may be more useful to examine patterns of activation in individual patients, in particular, with clinical groups such as DLD that show a progression to a more heterogeneous deficit profile with age.

Brown et al. (2014) developed a single subject methodology to examine data at the individual level, instead of relying on group averages. In an anatomically constrained magnetoencephalography (aMEG) study of DLD, they employed a novel technique to investigate the dynamic functional brain organization for semantic processing in a young adult with DLD, his two siblings and a group of normal language controls. Despite behavioral performance that was similar to the normal controls and the two normal language siblings, the participant with DLD evidenced an abnormal pattern of cortical activity. In a direct, vertex-wise comparison to the distribution of neural activity at all cortical locations and time points within the typically developing group using *z*-scores, Brown and colleague were able to demonstrate that the participant with DLD showed qualitative differences from the typical dynamic functional organization that agreed with qualitative comparisons of the dSPMs.

Functional near-infrared technology (fNIRS) is an excellent alternative to fMRI that is particularly suitable for spoken language studies with infants, children, and medically fragile populations. In particular, the portability of fNIRS systems allows for functional imaging studies to be conducted in optimal speech research environments. fNIRS is a neuroimaging tool using near-infrared light (i.e., 700–1000 nm) to non-invasively monitor the hemodynamic responses evoked by neuronal activity (Villringer and Chance, 1997; Strangman et al., 2002; Izzetoglu et al., 2011). It measures the quantitative change in oxygenated hemoglobin (HbO_2_) concentration and deoxygenated hemoglobin (Hb) concentration in the cerebral blood flow. To date, only one study has used fNIRS with DLD populations with the focus of the study on differences in patterns of brain activation during sentence comprehension (Fu et al., 2016). With respect to working memory, many studies have used fNIRS and an n-back working memory paradigm to examine frontal lobe processing during both encoding and updating phases of the task in both typical and clinical populations (e.g., Ehlis et al., 2008; Fishburn et al., 2014; Peck et al., 2014; Unni et al., 2015; Yeung et al., 2016; Berglund-Barraza et al., 2019a; Putze et al., 2019). Consistent with fMRI studies, fNIRS studies show that that both HbO_2_ and Hb are sensitive to changes in cognitive effort in working memory tasks in the frontal lobe (i.e., Fishburn et al., 2014; Unni et al., 2015; Yeung et al., 2016, 2018; Causse et al., 2017; Putze et al., 2019) as well as being sensitive to altered frontal activation patterns during working memory tasks in clinical populations such as attention-deficit disorder, and high-functioning autism spectrum disorder (Ehlis et al., 2008; Yeung et al., 2019), however, no studies have used fNIRS to characterize the frontal activation patterns in DLD during working memory (Butler et al., 2020).

As noted above, a small but growing body of work shows a pattern where performance on behavioral tasks appears to overlap with that of normal language controls, but the underlying cortical activation is abnormal in adolescents and young adults with DLD (Brown et al., 2014; Haebig et al., 2017). In a recent fNIRS study, we showed that a clinical marker of DLD may be the use of atypical cortical networks that do not result in a one-to-one link between performance and brain activation on verbal working memory tasks in DLD (Berglund-Barraza et al., 2019b ASHA poster). Using the same single-subject design as Brown et al. (2014), we found that fNIRS was sensitive to the presence of abnormal hemodynamic activation in an individual adult with DLD despite behavioral performance that resembled that of a group of age- and gender-matched normal language controls.

Because it is relatively user friendly and well suited to auditory processing studies that require quiet listening environments, in this study we used fNIRS to examine the hemodynamic response in the prefrontal lobe in individual subjects with DLD before and after phonological working memory training. To test the potential clinical benefits of fNIRS to document changes in the hemodynamic response in individual participants with DLD, we used a high-definition transcortical direct current stimulation (HD-tDCS) enhanced phonological working memory training paradigm developed in our lab (Berglund-Barraza et al., under review). In contrast to the broader concept of “working memory” that refers generally to a cognitive system that allows for the manipulation of stored information and which is supported by networks in the prefrontal cortex, in this study we focus on phonological working memory. Phonological working memory refers to the cognitive processes that underlie the temporary storage of phonological information (i.e., the sounds of language) in memory and prevents its decay through continuous refreshing via the rehearsal loop (i.e., Baddeley and Hitch, 1974).

Phonological working memory plays a key role in a wide range of language skills including, but not limited to, learning of novel words, vocabulary development, the maintenance and reactivation of information during sentence and discourse processing, the acquisition of reading abilities, and second language acquisition (Papagno et al., 1991; Adams and Gathercole, 1995; Adams, 1996; Baddeley et al., 1998). Phonological working memory deficits are a key feature in the DLD deficit profile (c.f. Ellis Weismer et al., 2000; Estes et al., 2007; Coady and Evans, 2008) and account for significant variance in lexical and sentence comprehension in both typically developing children and children with DLD where better phonological working memory correlates with better vocabulary and sentence comprehension abilities in children and adults with and without language disorders (Caplan and Waters, 1999; Alt, 2011; Gillam et al., 2019). Given the broad impact of these phonological working memory deficits, there has been a growing interest in improving the working memory abilities of individuals with DLD. In this study we extend this work to ask if this abnormal pattern of cortical activity coupled behavioral performance that falls within normal limits is evident in additional adults with DLD, and if fNIRS can track potential changes in the cortical networks engaged by adults with DLD following phonological working memory training.

Attempts to boost phonological working memory have directly targeted the rehearsal processes that maintain the representations of speech sounds in verbal short-term memory through overt rehearsal of items. Approaches focusing on overt rehearsal have met with limited success, in particular with clinical populations, and even when relatively modest gains have been observed, the gains have been found to persist for only short periods of time (i.e., Gathercole and Alloway, 2006). Neuroimaging studies using fMRI and EEG suggest the areas in the prefrontal cortex (PFC) are involved in working memory functions and concurrent use of tDCS and cognitive training has also been explored as a way to enhance verbal working memory, in particular with stimulation to the dorsolateral prefrontal cortex (DLPFC) concurrent with verbal working memory training (i.e., Andrews et al., 2011; Ruf et al., 2017). Importantly, however, these effects are evident only when tDCS stimulation in regions hypothesized to support working memory are coupled with working memory training at the time of stimulation (Hill et al., 2016; Mancuso et al., 2016).

In contrast to the PFC, the pre supplementary motor area (preSMA) appears to be a key part of the cortical network that supports phonological working memory in particular (Mecklinger et al., 2000; Linden et al., 2003; Marvel and Desmond, 2010; Cummine et al., 2017). The high connectivity between preSMA and brain regions associated with working memory, such as the prefrontal cortex and caudate (Zhang et al., 2012), and the preSMA’s response to cognitive load during productive language tasks requiring phonological working memory led us to focus stimulation of preSMA to enhance phonological working memory training (Berglund-Barraza et al., under review). This subsequently led to behavioral changes during active spoken word processing in typical language adults.

Given these benefits of tDCS, in this study we assess if fNIRS is also an effective method to document functional changes in cortical activity in response to HD-tDCS working memory training in a single subject with DLD. Transcortical direct current stimulation (tDCS) involves the application of small amounts of electrical current through electrodes placed on the scalp to modulate the underlying neural areas’ cortical excitability and activity (Nitsche and Paulus, 2000, 2001; Stagg and Nitsche, 2011; Moreno-Duarte et al., 2014; Biabani et al., 2017; Matsumoto and Ugawa, 2017). In contrast to traditional tDCS approaches, the use of High Definition tDCS (HD-tDCS) improves the spatial preciseness of traditional tDCS by utilizing a 4 × 1 ring electrode array to modulate a restricted cortical area (Alam et al., 2016). By improving upon the localized specificity of tDCS, HD-tDCS enables researchers to ask pointed questions about the behavior of specific brain regions. Because anodal HD tDCS is thought to bring neurons closer to their firing threshold, efforts to improve working memory have begun including the use of anodal HD tDCS stimulation to areas necessary for working memory function, with varying results in neurotypical populations.

In this study, we asked if fNIRS ability to identify the potential use of abnormal cortical networks in a single adult subject with DLD during n-back working memory task extends to additional adults with DLD, and if fNIRS can track possible changes in the networks used by adults with DLD following intervention to the phonological working memory system enhanced with anodal HD tDCS stimulation to the preSMA and the circuits believed to support phonological working memory (i.e., preSMA).

## Materials and Methods

### Participants

Participants consisted of two adult females with a documented history of DLD (age 25 years) and twenty-one age- and gender-matched (ages 18–25 years) normal controls. All participants in the study were college students at the University of Texas Dallas. All were female, were strongly right hand dominant, and were monolingual speakers of English. None of the participants had a history of neurological injury or disease, seizure disorder, or psychiatric diagnosis, and none of the participants reported using psychotropic medications such as stimulants or anti-depressants.

The two participants with DLD both received formal diagnosis of language learning disabilities in elementary school, received speech, and language services throughout elementary school and middle school, and qualified for and received academic accommodations for language learning disabilities as an undergraduate and graduate student. Standardized language assessment at the time of the study confirmed the continued presence of DLD in both participants (Fidler et al., 2011).

The participants in the normal control group all reported a history of normal language development, no diagnosis of speech, language or learning disorders and no special education services at any point in time. All participants completed written informed consent protocols in accordance with the Declaration of Helsinki as well as the guidelines of the University of Texas at Dallas Institutional Review Board (IRB), which approved the protocol. Participants received financial compensation or college credit for their participation in the study.

### Study Design

**FIGURE 1:**
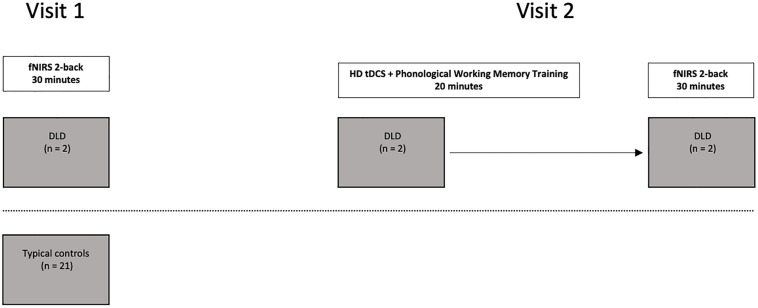
Study design outline for the participants with DLD (*n* = 2) and typical controls (*n* = 21).

To examine the hemodynamic response during active spoken word processing during verbal working memory, all the participants completed an auditory 2-back working memory task using the fNIRS protocol outlined in Berglund-Barraza et al. (2019a). The two participants with DLD then completed a phonological working memory training protocol enhanced with anodal HD tDCS stimulation to the preSMA. Following the HD tDCS training, the two participants with DLD then completed the auditory 2-back working memory task a second time. Both participants with DLD completed the auditory 2-back working memory task a second time immediately following completion of the HD tDCS phonological working memory training protocol (see Figure 1). For DLD-1 this occurred 6 months after the initial completion of the auditory 2-back working memory task and for DLD-2 this occurred 2 weeks after completing the auditory 2-back working memory task for the first time.

#### fNIRS Working Memory Task

In the task, the participants heard a series of spoken words and were asked to press a button only when the word they heard was *“the same as the word”* they heard two back in the sequence. Using a block design, participants completed 14 blocks consisting of seven different fixed random-order sequences of the match and non-match trials All blocks contained a total of 98 words and the proportion of target matches (25%) was the same across all of the blocks, with no more than three consecutive match trials occurring in a row in each block. Half of the fourteen blocks consisted of words having high word frequency + high phonotactic probability (the “HF” blocks) and the other seven blocks consisted of words low word frequency + low phonotactic probability (the “LF” blocks). The neighborhood density of the words in all of the blocks were high and did not differ across the high- and low-word frequency blocks. For detailed description of the task stimuli see Berglund-Barraza et al. (under review). Using a conventional fMRI block design, HF and LF blocks were presented in an alternating manner beginning with a HF block.

Participants sat in front of a computer screen in a sound attenuated, dark room. Stimuli were presented binaurally via foam insert earphones at a comfortable listening level. To ensure participants understood the task, participants were first trained on 22 visual practice trials (clip art color pictures of common animals and objects (e.g., *pig, tree, stove, lamp, etc.*) and then completed 22 auditory practice trials. Participants complete all practice trails correctly before continuing onto the experimental task.

The stimuli consisted of one-syllable spoken real words digitally recorded at 44.1 Hz by an adult female native speaker of English. Each word was approximately 700-milliseconds in duration and each sound file contained an initial 50 milliseconds of silence and each word began with a 5-millisecond envelope. Silence was added at the end of each sound file so that all files were 1000-milliseconds in duration and words were presented with an interstimulus interval (ISI) of 500 milliseconds resulting in each block being 90 s in duration. Each block was separated by a 15 s resting state period of silence where the participants were instructed to take a break and not move. The words in the HF and LF blocks differed in both whole word frequency and sublexical phonotactic frequency [word frequency, *F*(1,13) = 310.5, *p* < 0.001; biphone phonotactic frequency, *F*(1,13) = 9877, *p* < 0.001]. Because the inhibitory effect neighborhood density can the effect of sublexical phonotactic frequency, in part because the two measures are highly correlated (i.e., Coady et al., 2010), all of the words in both conditions had low neighborhood density with ratings at or below 20 and neighborhood density did not differ for the HF and LF conditions, *F*(1,12) = 0.01 and *p* = 0.92. Imageability was also controlled, with all of the words in both conditions having imageability rating of 5.0 or higher (Walker and Hulme, 1999) and imageability did not differ for HF and LF conditions, *F*(1,13) = 0.29 and *p* = 0.60.

#### Anodal HD tDCS Enhanced Phonological Working Memory Training

Using the same anodal HD tDCS phonological working memory enhanced training protocol outlined in Berglund-Barraza et al. (under review). While undergoing anodal HD tDCS, the participants with DLD completed a phonological working memory training task where they overtly repeated a series of non-words (4–7-syllables in length) presented in a random order. The non-words consisted of recordings of one adult female with a standard English dialect, digitally recorded and presented over speaker in a quiet room. The non-word list comprised 120 of non-words taken from the Gupta et al. (2004) study, and were controlled for syllable length, onset consonant, and phonotactic probability. All non-words were composed of English consonants and vowels, with syllable combinations following standard American English phonotactic rules.

### Functional Near Infrared Spectroscopy

**FIGURE 2:**
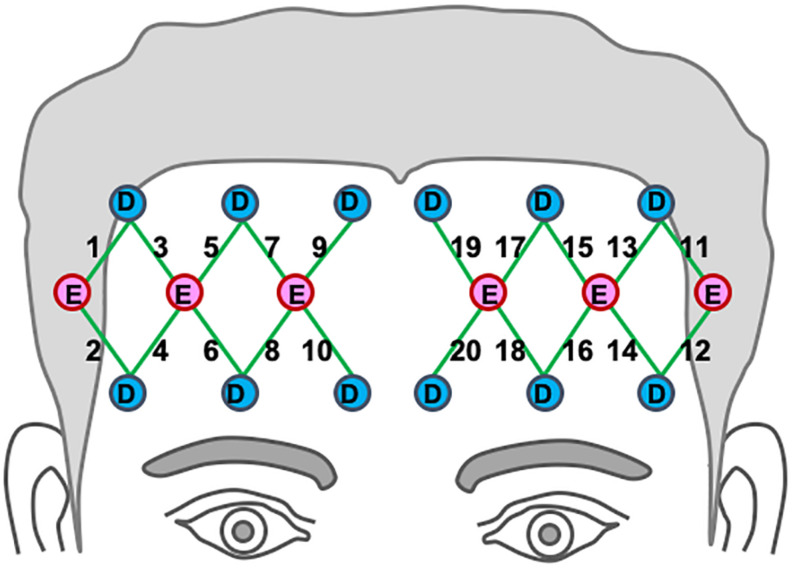
Location of optodes across the participants’ bilateral prefrontal cortex. Detectors are marked as “D” in blue circles, and emitters are marked “E” in red circles. Channel paths are represented in the 20 green lines (Berglund-Barraza et al., 2019a).

A continuous-wave, multi-channel fNIRS system was used to acquire hemodynamic activities in the prefrontal region (TechEn, Inc., Milford, MA, United States). The system uses near-infrared lasers at 690 and 830 nm as emitters, and avalanche photodiodes as detectors. A fiber optic probe that contained 6 emitting optodes and 12 detecting optodes was placed bilaterally and symmetrically on the participant’s forehead, as shown in Figure 2. The probe was secured in place with an elastic band during the experiment. The bottom of the probe array was placed just above the eyebrows. This optode montage provides 20 channels of hemodynamic measurements at a constant emitter-detector distance of 3 cm. Areas underlying the channels are approximately over Brodmann’s areas 10 and 46. The data sampling rate was 25 Hz.

#### fNIRS Data Processing and Artifact Removal

The fNIRS data were processed using Homer software (Huppert et al., 2009). The raw data were band-pass filtered between 0.005-0.2 Hz to remove slow drifts and high-frequency noises, and then converted into changes in optical density. Blocks corrupted by large motion artifacts (e.g., yawns, coughs) were manually removed. Changes in oxygenated hemoglobin (HbO_2_) and deoxygenated hemoglobin (Hb) concentrations were calculated based on the Modified Beer-Lambert Law (Baker et al., 2014) with a partial path-length factor of 6.0 for both wavelengths. At last, block-averaged HbO_2_ and Hb responses for each of the 20 channels were derived from the motion-free blocks, which were then used to reconstruct topographic images of prefrontal activation based on default Homer functions.

### High Definition Transcranial Direct Current Stimulation (HD tDCS)

The participants with DLD underwent 20 min of anodal HD tDCS stimulation to the preSMA. Quik-Gel Conductive Gel was inserted under each electrode to make contact with the scalp (3.1 cm2 per electrode). The participants wore a cap with five electrodes inserted into it, one anode (Fz) and four return cathodes (Fpz, F7, F8, and Cz), placed according to the 10-10 EEG system for electrode positioning (Starstim, Neuroelectrics, Barcelona, Spain). Current ramped up over 30 s and maintained a continuous current for the entire 20 min. For anodal stimulation, a wireless battery driven multi-channel direct current stimulator (Starstim, Neuroelectrics, Barcelona, Spain) delivered a direct current over the scalp. This design allows for focal delivery of anodal current to the preSMA, using a constant current of 1.0 mA while applying weaker cathodal current. The stimulation montage was developed to target the preSMA by generating a model of the electric field distribution for the configured stimulation protocol with Neuroelectrics Instrument Controller software (Barcelona, Spain).

### Statistical Analysis

#### Behavioral Performance

Participants’ accuracy on the task was measured using *d’*, a sensitivity index in *z* units, that differentiates the means for the signal (i.e., target) versus noise (i.e., foils) and is a direct measure of the listener’s ability to recognize/differentiate target from foil trial while controlling for listener sensitivity and response bias (MacMillan and Kaplan, 1985). Perfect performance on the task was *d*’ = 8.6. In addition to overall accuracy, the *n*-back design allows for detailed analysis of two different response types: (1) HITs, where the participant correctly recognized a target, which is a measure of the participant’s ability to hold the target word in memory, and (2) Correct Rejections (CR), a measure of the participant’s ability to inhibit lexical interference from those words that did not match (Qin et al., 2016). Reaction time (RT) was analyzed for correct trials with no trimming of outliers. To determine the degree to which the performance of the participants with DLD fell outside of the normal range at pre- and post-treatment, *z*-scores were calculated using the normal controls’ performance as the normative database in the same manner as that described by Brown et al. (2014).

#### Hemodynamic Response

For the normal controls, channel-wise mean hemodynamic changes were calculated for each subject and compared to the resting state for the HF and LF conditions separately. Individual *t*-tests were then conducted for each channel to identify those regions of interest (ROIs) where hemoglobin levels differed significantly from resting state for each condition. To control for the inherent diffusive characteristics of fNIRS imaging, data was collapsed to create a single ROI for those regions where hemoglobin levels differed significantly from rest in spatially adjacent channels within hemisphere. To determine the extent to which the hemodynamic response for the DLD participants fell within/outside normal limits prior to and following HD tDCS training z-scores were calculated based on the normal control data. In characterizing the hemodynamic response for the DLD participant, standardized norm referenced z-scores for changes in HbO2 and Hb from resting state were first calculated using the ROIs derived from the control group to determine the extent to which the hemodynamic response for the DLD participants were similar/differed from that of the normal controls. To determine the extent to which the hemodynamic response for the DLD participants *differed qualitatively* from normal, channel-wise standardized norm referenced z-scores were then calculated from the normal control data.

## Results

### Behavioral Performance

#### DLD-1

Using ± 1 *SD* cut-off, pre-treatment, *d*’ *z*-scores for the DLD-1 was below age- and gender-matched expectations for HF words (*z* = −1.36) but within normal limits (WNLs) for LF words (*z* = 0.34)^1^. Post-treatment, her performance improved for HF (*z* = −1.13), and LF words remained WNL (*z* = 0.07). Although *z*-scores for RT for DLD-1 were WNLs prior to training (HF: *z* = 0.59; LF *z* = 0.56) they also remained WNL post treatment (HF: *z* = 0.07; LF: *z* = 0.53). As can be seen in Table 1, DLD-1’s poor performance in both the HF and LF conditions prior to training was due to low HIT rates as compared to her peers, indicating that she had significant trouble holding and subsequently recognizing the target word in auditory working memory (i.e., HITs), but did not have trouble inhibiting interference from lexical competitors (i.e., Correct Rejections). Following HD tDCS enhanced phonological working memory training, DLD-1’s performance improved on behavioral performance measures as can be seen in her increased ability to hold words in memory (i.e., better HIT *z*-scores), and an increase in her ability to inhibit interference from competitors (i.e., better Correct Reject *z*-scores) for both HF and LF words. Taken together, for DLD-1, behavioral performance changes pre- post-treatment whereas her speed of responding did not.

**TABLE 1:** Z-scores for hits and correct rejects pre- and post-training for DLD-1 and DLD-2.

		High-frequency		Low-frequency	
		Hit	Correct reject	Hit	Correct reject
DLD-1	Pre-treatment	−1.30^†^	–0.12	−1.15^†^	–0.15
	Post-treatment	0.20	1.10^†^	0.07	0.87
DLD-2	Pre-treatment	0.45	1.11^†^	0.15	0.70
	Post-treatment	0.45	0.61	0.53	0.70

*^†^ ≤ 1.0 SD, ≥1.0 SD.*

#### DLD-2

Using ± 1 *SD* cut-off, prior to training, *d*’ *z*-scores for the DLD-2 was within age- and gender-matched expectations for HF words (*z* = 0.71) and LF words (*z* = 0.57). Following HD tDCS enhanced phonological working memory training, her performance still fell WNLs for HF words (*z* = 0.12) and LF words (*z* = 0.70). Prior to training, DLD-2’s *z*-scores for RT were WNLs for HF words (*z* = −0.42), but fell below normal limits for LF words (*z* = −1.02). Following the HD tDCS combined phonological working memory training, DLD-2’s performance improved for both HF and LF words with RT z-scores falling WNLs for *both* HF words (*z* = 0.56) and LF words (*z* = −0.69). Standardized *z*-scores for HITS and CR for DLD-2 are shown in Table 1. As can be seen in Table 1, DLD-2’s performance was notably stable between pre- and post-treatment. In contrast to DLD-1, the speed/accuracy trade-off pattern differed for DLD-2. In particular, while her behavioral performance did not change appreciably pre- to post-treatment her speed of responding did, indicating that she was faster to correctly process the information.

### Hemodynamic Response in the Prefrontal Cortex

#### Normal Controls

**FIGURE 3:**
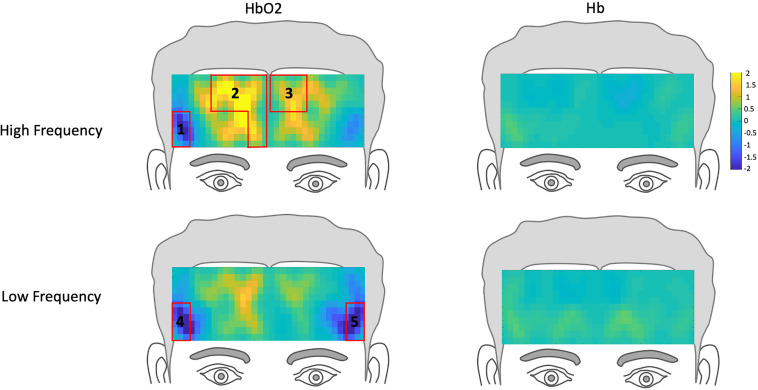
Hemodynamic topography of activation in the prefrontal cortex for normal controls, including ROIs outlined in red. HbO_2_ and Hb for high-frequency condition with ROIs 1-3, and HbO_2_ and Hb for low-frequency condition, with ROIs 4 and 5.

To examine the extent to which DLD participant’s hemodynamic response differed from that of the normal controls, we compared their hemodynamic response to those ROIs where activation was significantly different from rest in the normal controls for HF and LF words. The channels where the hemodynamic response was significantly different from rest in the frontal lobe for the normal control group are shown in Table 2 and Figure 3. Similar to our prior study (Berglund-Barraza et al., 2019a), for the normal controls, for HF words, the hemodynamic response was significantly different from rest for three ROIs. This hemodynamic response was characterized specifically by a significant *decrease* in HbO2 (i.e., suppression) of a right lateralized inferior region (ROI1 from channel 2) and a significant increase in HbO2 response in medial pre-frontal region (ROI2 from channels 5,7,9,10) and superior frontal region (ROI3 from channels 17 and 19). In the LF word condition, HbO2 response was significantly different from rest for the normal controls in two ROIs. Like the HF condition, there was a significant decrease in activation (i.e., suppression) of the same right lateralized inferior region as seen for HF words (ROI1 from channel 2). However, unlike HF words, there was no increase in pre-frontal activation (i.e., ROI2 and ROI3). Instead there was also a significant decrease in activation (i.e., suppression) in the left lateralized inferior region (ROI5 from channel 12), which was not evident in the HF condition and is contralateral to ROI1 (HF) and ROI4 (LF).

**TABLE 2:** HbO_2_ and Hb *t* and *p* statistics for high frequency (HF) versus resting, and low frequency (LF) versus resting conditions for the normal controls.

Channel	HF vs. Resting				LF vs. Resting			
	HbO_2_		Hb		HbO_2_		Hb	
	*t*	*p*	*t*	*p*	*t*	*p*	*t*	*p*
1	–0.61	0.55	0.15	0.88	–1.19	0.25	1.15	0.26
2	–2.40	0.03*	1.29	0.21	–3.53	0.00**	1.39	0.18
3	1.92	0.07	–0.96	0.35	0.89	0.38	–0.63	0.54
4	–0.09	0.93	1.07	0.29	–1.05	0.31	0.77	0.45
5	2.66	0.02*	–0.27	0.79	0.81	0.43	0.24	0.81
6	1.28	0.22	0.67	0.51	0.42	0.68	0.55	0.59
7	3.11	0.01**	–0.60	0.55	1.25	0.23	–0.86	0.40
8	1.95	0.07	0.68	0.51	0.53	0.60	1.35	0.19
9	2.87	0.01**	0.46	0.65	1.93	0.07	–0.32	0.76
10	2.41	0.03*	0.34	0.74	1.09	0.29	1.70	0.11
11	0.81	0.43	0.16	0.87	–1.60	0.13	0.68	0.51
12	–1.78	0.09	1.01	0.33	–2.63	0.02*	1.26	0.22
13	1.00	0.33	0.88	0.39	0.06	0.95	0.11	0.91
14	–0.04	0.97	–0.03	0.97	–1.57	0.13	0.13	0.89
15	2.04	0.06	–0.17	0.87	0.32	0.75	0.19	0.85
16	1.17	0.26	–0.06	0.95	–0.46	0.65	0.96	0.35
17	2.77	0.01**	–1.19	0.25	1.38	0.18	0.33	0.75
18	1.95	0.07	0.12	0.91	0.52	0.61	1.89	0.07
19	2.38	0.03*	–1.06	0.30	1.78	0.09	–0.57	0.58
20	1.08	0.29	–0.00	0.99	–0.12	0.91	1.88	0.08

*p < 0.05, **p < 0.01.

##### fNIRS pre-treatment

**FIGURE 4:**
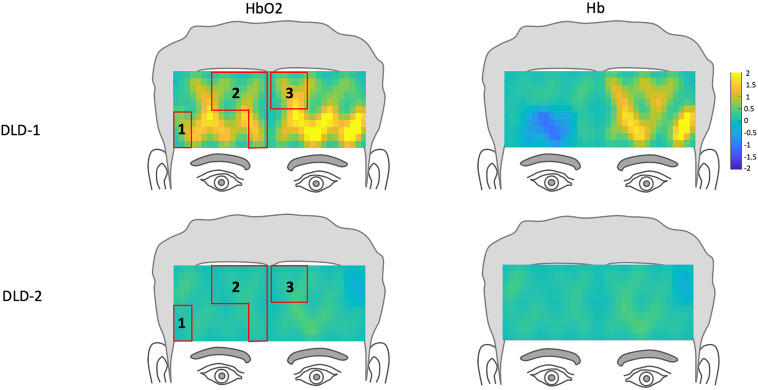
*Z*-score topographs for DLD-1 and DLD-2 from pre-training, for high-frequency words for HbO_2_ and Hb. ROIs established by the normal controls are outlined in red.

**FIGURE 5:**
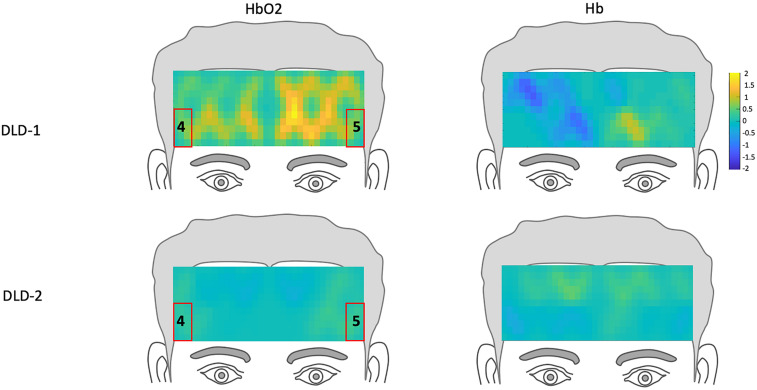
*Z*-score topographs for DLD-1 and DLD-2 from pre-training, for low-frequency words for HbO_2_ and Hb. ROIs established by the normal controls are outlined in red.

The *z*-scores topographs for changes in hemodynamic response pre-treatment for DLD-1 and DLD-2 for HF are shown in Figure 4 and for LF in Figure 5. For DLD-1, prior to HD tDCS enhanced phonological working memory training, for HF words, her hemodynamic response was significantly above age- and gender expectations for all three ROIs. For DLD-2, her hemodynamic response was WNL for all three ROIs. For LF words, for DLD-1, her hemodynamic response was significantly outside the normal range for ROI4 but WNL for ROI5. Standardized z-scores for changes in hemodynamic response pre-treatment for DLD-2 for LF words were significantly outside the normal range for ROI4 but WNLs for ROI5.

##### fNIRS post-treatment

**FIGURE 6:**
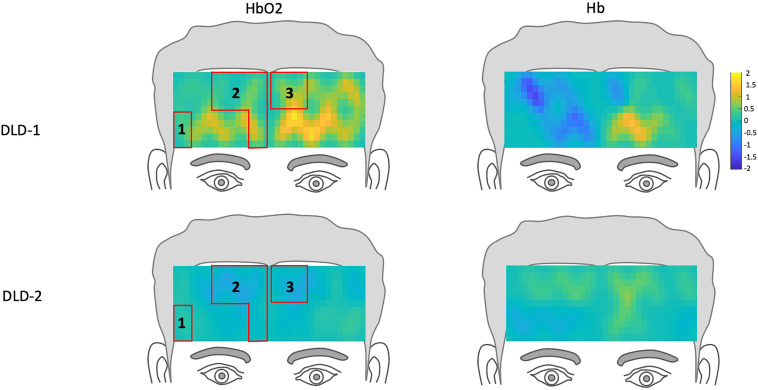
*Z*-score topographs for DLD-1 and DLD-2 from post-training, for high-frequency words for HbO_2_ and Hb. ROIs established by the normal controls are outlined in red.

**FIGURE 7:**
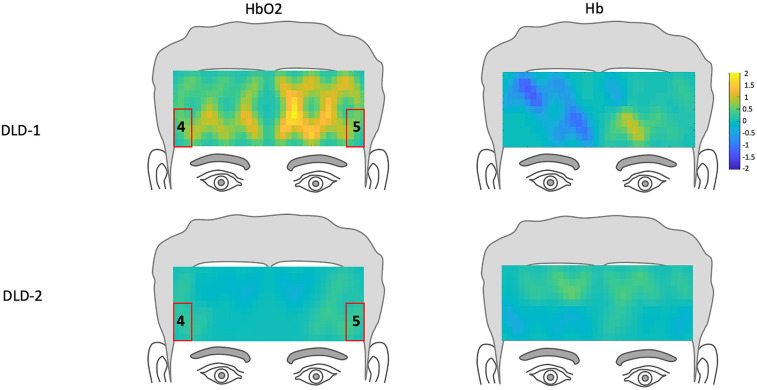
*Z*-score topographs for DLD-1 and DLD-2 from post-training, for low-frequency words for HbO_2_ and Hb. ROIs established by the normal controls are outlined in red.

The channel-wise, block-averaged z-scores topographs for DLD-1 and DLD-2’s hemodynamic changes for HF and LF conditions post-treatment are shown in Figure 6 for HF words and Figure 7 for LF words. For DLD-1, post HD tDCS enhanced phonological working memory training, hemodynamic response in ROI 1 and ROI 2 fell WNL and was outside normal limits for ROI 3. For DLD-2, her hemodynamic response was WNL for all three of the HF ROIs. For LF words, both DLD-1’s and DLD-2’s hemodynamic responses were WNL for both ROIs.

The changes in *z*-scores for changes in hemodynamic response from resting state pre- to post-treatment for HF and LF conditions for DLD-1 and DLD-2 are also shown in Table 3. As can be seen in Table 3 and Figures 3–6, the single-subject design and fNIRS protocol used in this study enabled us to characterize the degree to which the hemodynamic responses in the DLD participants for the ROIs engaged by normal controls was abnormal or not. Importantly, this design also enabled us to identify individual differences in each DLD’s response to the phonological working memory training with respect to both changes in behavioral performance and the degree to which their hemodynamic responses shifted to fall more in line with that of the normal controls post-treatment.

**TABLE 3:** Pre-training and post-training HbO_2_ *z*-scores for high- and low-frequency for DLD-1 and DLD-2.

		High-Frequency			Low-Frequency	
		ROI 1	ROI 2	ROI 3	ROI 4	ROI 5
DLD-1	Pre-training	1.21^†^	1.56^†^	1.63^†^	1.34^†^	0.82
	Post-training	0.57	0.91	1.10^†^	0.83	0.90
DLD-2	Pre-training	0.31	0.42	0.51	1.24^†^	0.38
	Post-training	0.27	–0.45	–0.68	0.44	0.65

*^†^ ≤ 1.0 SD, ≥ 1.0 SD.*

## Summary of Results and Conclusion

This study used fNIRS to examine prefrontal lobe hemodynamic response during an active, spoken word processing task in two adults with DLD prior to and following an HD tDCS enhanced phonological working memory training task using the behavioral and hemodynamic response data from a group of age- and gender-matched normal language controls as the normative sample. Specifically, we asked if fNIRS can be used to document changes in individual hemodynamic response at the single subject-level pre- and post-treatment. The results from this study showed significant differences in hemodynamic response between the two DLD participants, and additionally showed differences between hemoglobin levels between the participants with DLD and normal controls, despite near-normal behavioral performance for DLD-1, and completely WNL behavioral performance for DLD-2. Additionally, the results showed stark differences in cortical response across the PFC for both participants with DLD pre- and post-HD tDCS enhanced phonological working memory training.

Taken together the findings from this study show that the fNIRS protocol that was used in this study was able to characterize qualitative differences in the hemodynamic response pattern in individual subjects with DLD in the prefrontal lobe during a working memory task. For DLD-1, looking just at her behavioral performance in the auditory working memory task alone, her overall performance is consistent with prior studies showing that individuals with DLD are sensitive to word frequency effects (Mainela-Arnold and Evans, 2005; Mainela-Arnold et al., 2008). However, her hemodynamic response was atypical as compared to normal controls. Together these data fall in line with prior studies also suggesting overlap in behavioral performance combined with atypical cortical response. Following training, fNIRS was able to detect changes in her hemodynamic response, that was characterized predominantly as a shift more toward that of the normal controls.

We hypothesize that a clinical marker of young adults with DLD may be a decrease in activation of task-specific brain regions used by normal controls, coupled with increased activation of prefrontal areas that have been interpreted as maladaptive overactivations in normal aging, when compared to young adults, in task of executive functions (Basak et al., 2018; Nashiro et al., 2018) and working memory, including a 2-back task (O’Connell and Basak, 2018). The findings from this study showed the fNIRS can be used in a single subject design to compared the hemodynamic response of clinical populations to that of normal controls, but also to document changes in hemodynamic response in clinical populations following treatment in a manner that is more sensitive than what can be ascertained from behavior performance alone. That participants with DLD’s hemodynamic response dropped to WNLs for *nearly all* ROIs post-HD tDCS enhanced phonological working memory training suggests that following training they were able to perform the same task but with different cortical networks.

Future research could utilize fNIRS and the single-subject methodology outlined in this study to extend the research on maladaptive activation found in normal aging to investigate the cortical activation differences between typical and atypical young adult populations during spoken language tasks. To date, the majority of DLD imaging studies have used designs that compare a group of individuals with DLD to a control group. As a simple illustrative example of the limitations of using group designs in imaging DLD studies is as follows: if in a group of nine individuals with DLD, three individuals show normal brain activity in the three ROIs for HF words as the normal controls, three show over activation in the three ROIs, and three show under-activation in these three ROIs, the group-averaged statistical parametric map of activity for the nine DLD subjects will appear WNLs, thus qualitatively misrepresenting two-thirds of the individual subjects. In addition, the high individual variability in functional organization in DLD, in direct comparison with control subjects, would prevent corrected statistical tests of a group difference from reaching significance. This type of group analysis, performed without examination of individual data would lead the researchers to mistakenly conclude that individuals with DLD has a similar functional organization to normal controls as they would appear show no significant differences in functional organization from TD controls (two conclusions commonly reached in published fMRI, EEG, and MEG studies). Hence, the need for the development of methodological and conceptual approaches for the use of functional neuroimaging to assess individual differences in the brain-behavior relationship in DLD. This study highlights the additional knowledge that can be gained from examining individual differences in patterns of brain activation in clinical groups such as DLD that have a high degree of heterogeneity in some aspects of the deficit profile.

Importantly, the fNIRS protocol used in this study was also sensitive to differences at the individual subject level, as evidenced by the pattern seen in DLD-2 which differed from that of DLD-1. Specifically, behavioral performance for DLD-2 fell WNLs both prior to training and following training. Similarly, for DLD-2, her hemodynamic response was largely similar to that of the normal controls both prior to training and following training. A key, yet still unanswered, question in the field is whether DLD comprises a distinct clinical disorder or merely represents those individuals who are otherwise normal but whose language abilities simply fall at the low end of the normal distribution (Leonard, 1987). Similarly, data suggest that the language deficits for some individuals diagnosed with DLD as children may resolve in adulthood. Theoretically, the findings from this study have clinical implications in that they suggest that combined behavioral and fNIRS data may be valuable in future studies designed to determine the extent to which DLD has resolved or is still present in individual adults with a documented history of DLD as children.

Additionally, the results showed that the fNIRS protocol used in this study was sensitive to the effects of the anodal HD tDCS enhanced phonological working memory training. Specifically, for both participants with DLD there was evidence of a change in the hemodynamic response for both HF and LF words post-treatment. In some cases, the hemodynamic response for both participants moved to fall further within the 1 *SD* cut off following training, but interestingly, the protocol was also able to detect hemodynamic response that moved closer to fall outside the 1 *SD* cutoff. In this study we used ± 1SD as our cut-off in keeping with the DSMD-5 (American Psychiatric Association, 2013). Although this was still an arbitrary cut-off in some respects, it highlights the strength of using z-scores, regardless of the cut-off, to provide a more nuanced measure of the severity or degree to which the pattern of activation for the DLD participants was atypical as compared to the normal controls.

While intriguing, there are limitations with the study’s methodology that warrant caution with attributing any behavioral or neurological changes directly to the application of HD-tDCS. Several substantive issues and limitations in this study warrant further discussion. First, one question is whether the changes in both the behavioral performance and hemodynamic levels we observed for both the participants with DLD were the result of the training protocol or simply due to task practice. In a second study (Berglund-Barraza et al., under review), we directly investigated, in a group of typical adults, the potential added influence of HD tDCS stimulation to preSMA to the phonological working memory training protocol used in the current study. We found a statistically significant change in participants’ phonological working memory following training when combined with HD tDCS stimulation (i.e., active) as compared to phonological working memory training without stimulation (i.e., sham). Understanding the nature of neurobiology of DLD in adults may require methods that can examine the relationship between behavioral performance and cortical activation patterns in these individuals. While it is entirely possible that the changes we observed in the two participants in the current study were been due to practice effects and not the HD tDCS stimulation and/or phonological working memory training, the findings from this study indicate that fNIRS appears to be a valuable tool that can be used in future research both being to characterize the neurobiology of adults with DLD, but also to investigate the feasibility of various therapeutic interventions.

In tDCS studies, training typically includes multiple sessions (Au et al., 2016; Ruf et al., 2017; Ke et al., 2019). For example the participants in Ruf et al. (2017) received tDCS stimulation concurrent with working memory training over a period of three training sessions whereas the participants in Richmond et al. (2014) tDCS enhanced working memory training over a period of 10 training sessions. Similarly, studies that have focused on improving working memory in individuals with and without DLD also include multiple sessions (Stanford et al., 2019). Although the number of sessions varies across studies, a consistent finding in working memory training studies is that the programs are effective as a tool in producing reliable short-term improvements in working memory skills in individuals with cognitive disorders (i.e., DLD, ADHD, etc.), Melby-Lervåg and Hulme (2013). In this study, we use the term “training” to refer to the 20 min that the DLD participants spent completing the non-word repetition task that was combined with the HD tDCS stimulation to the preSMA. Specifically, we examine whether this very short duration phonological working memory training session enhanced with HD tDCS would result in changes in hemodynamic response in the frontal lobe for these two participants. The results from this study indicate that as little as 20 min of phonological working memory training resulted in a frontal lobe hemodynamic response for the DLD participants that resembled that of the normal controls. The findings from this study raise the possibility that this shorter protocol might be an effective tool when used in conjunction with traditional therapy where the client receives a very short session of HD tDCS stimulation combined with working memory training prior to the therapy session to induce short term improvement in working memory that would enhance the therapy session. For example, results from a recent large-scale study indicate that phonological working memory is a major factor in the successful comprehension of both simple and complex spoken sentences in both individuals with and without DLD (Montgomery et al., 2018). The findings from the current study suggest that therapy sessions with individuals with DLD that are focused on improving comprehension of complex grammatical structures might benefit from the use of this HDtDCS phonological working memory protocol prior to the therapy session.

Taken together the results from this study demonstrate the feasibility and utility of using fNIRS to characterize individual differences in the cortical dynamics of clinical populations that may be associated with the interaction between language and cognitive processing. Further, the results from this study highlights the strength of fNIRS to characterize within subject changes in hemodynamic response following clinical training, at least over a short period of time. The findings for this study also suggest that fNIRS has the potential to be an invaluable tool to measure changes in cortical activation prior to and following intervention in future larger scale studies as well that examine therapeutic effects of brain training (i.e., tDCS) where participants are randomly assigned to active and sham conditions. Scientifically and clinically, there are many reasons to develop better functional neuroimaging methods to characterize single patients, not the least of which is differential diagnosis and development of more individualized treatment plans. However, technical, methodological, and conceptual barriers such as the weak signal-to-noise characteristics of the brain activity measures taken from only one patient, coupled with limited statistical approaches that can be adopted for making probabilistic comparisons and hypothesis tests based on data from a single-subject have limited progress in single subject studies. Additionally, due to sensitivity to movement artifacts and scanner noise, many neuroimaging techniques are unsuitable to investigate spoken language studies. Finally, unlike other brain imaging techniques, a unique aspect of fNIRS is that it allows the continuous assessment of changes in brain activation patterns both in real-time during experiments as well as across multiple experimental training sessions, in studies examining the relationship between brain and behavior for language comprehension and production.

## Data Availability Statement

The raw data supporting the conclusion of this article will be made available by the authors, without undue reservation.

## Ethics Statement

The studies involving human participants were reviewed and approved by the University of Texas at Dallas Institutional Review Board (IRB). The patients/participants provided their written informed consent to participate in this study.

## Author Contributions

JE, AB-B, FT, and JH designed the experiments. JE and AB-B developed the stimuli. FT, AB-B, and JE collected the fNIRS data, and processed and analyzed the data. AB-B and JE wrote and edited the manuscript with input from FT, CB, and JH. All authors contributed to the article and approved the submitted version.

## Conflict of Interest

The authors declare that the research was conducted in the absence of any commercial or financial relationships that could be construed as a potential conflict of interest.

## Footnotes

1 ±1 *SD* was used as our definition of language disorder consistent with the Diagnostic and Statistical Manual of Mental Disorders–Fifth Edition (American Psychiatric Association, 2013) definition of language disorder.
